# Epidemiology, Comorbidities, and Prescription Patterns of Korean Prurigo Nodularis Patients: A Multi-Institution Study

**DOI:** 10.3390/jcm11010095

**Published:** 2021-12-24

**Authors:** Yu-Ri Woo, Sehee Wang, Kyung-Ah Sohn, Hei-Sung Kim

**Affiliations:** 1 Department of Dermatology, Incheon St. Mary’s Hospital, The Catholic University of Korea, Seoul 06591, Korea; w1206@naver.com; 2 Department of Artificial Intelligence, College of Information Technology, Ajou University, Suwon 16499, Korea; wsh0509@ajou.ac.kr

**Keywords:** prurigo nodularis, epidemiology, comorbidities, prescription pattern, Korea

## Abstract

Prurigo nodularis (PN) is a chronic dermatosis typified by extraordinarily itchy nodules. However, little is known of the nature and extent of PN in Asian people. This study aimed to describe the epidemiology, comorbidities, and prescription pattern of PN in Koreans based on a large dermatology outpatient cohort. Patients with PN were identified from the Catholic Medical Center (CMC) clinical data warehouse. Anonymized data on age, sex, diagnostic codes, prescriptions, visitation dates, and other relevant parameters were collected. Pearson correlation analysis was used to calculate the correlation between PN prevalence and patient age. Conditional logistic regression modeling was adopted to measure the comorbidity risk of PN. A total of 3591 patients with PN were identified at the Catholic Medical Center Health System dermatology outpatient clinic in the period 2007–2020. A comparison of the study patients with age- and sex-matched controls (dermatology outpatients without PN) indicated that PN was associated with various comorbidities including chronic kidney disease (adjusted odds ratio (aOR), 1.48; 95% confidence interval (CI), 1.29–1.70), dyslipidemia (aOR, 1.88; 95% CI, 1.56–2.27), type 2 diabetes mellitus (aOR, 1.37; 95% CI, 1.22–1.54), arterial hypertension (aOR, 1.50; 95% CI, 1.30–1.73), autoimmune thyroiditis (aOR, 2.43; 95% CI, 1.42–4.16), non-Hodgkin’s lymphoma (aOR, 1.95; 95% CI, 1.23–3.07), and atopic dermatitis (aOR, 2.16, 95% CI, 1.91–2.45). Regarding prescription patterns, topical steroids were most favored, followed by topical calcineurin inhibitors; oral antihistamines were the most preferred systemic agent for PN. PN is a relatively rare but significant disease among Korean dermatology outpatients with a high comorbidity burden compared to dermatology outpatients without PN. There is great need for breakthroughs in PN treatment.

## 1. Introduction

Prurigo nodularis (PN) is a skin condition characterized by extremely pruritic and hyperkeratotic nodules on the extremities and body [1]. While vigorous scratching is an important trigger [2], the etiology of PN remains unclear.

PN is frequently linked with a personal history or atopy and can present concurrently with atopic dermatitis [3], with some recognizing it as a subtype of atopic dermatitis [4]. However, given that multiple pruritogenic diseases are associated with the emergence and resolution of PN upon treatment, it is more likely that the cause of PN is diverse [5].

PN was assigned a distinct International Classification of Diseases, 10th Revision (ICD-10) code in 2015 [6,7]. To date, the United States (US), Poland, and Germany have reported nationwide annual prevalence of PN with values of 6.52 (Poland) [8], 40 (Germany) [9], and 72 (US) [10] per 100,000 people. Ethnic differences likely exist, with PN disproportionately affecting African Americans [11].

PN patients often carry a significant disease burden with a higher risk of psychiatric disorders (anxiety, mood disorder) and systemic illnesses (chronic kidney disease) compared to the general population as well as those with other inflammatory skin diseases [11,12,13,14]. Of note, the association between HIV infection and PN was stronger in African Americans compared to a Caucasian cohort [11], which emphasizes the possible racial difference in PN co-morbidities.

The code for PN first appeared in the Korean Classification of Diseases (KCD) in 1993, 22 years prior to its introduction in ICD-10. Despite this early recognition, studies on PN in Koreans are almost nonexistent. Therefore, to gain a better understanding of this enigmatic condition, we performed a cross-sectional study on the epidemiology, demography, and co-morbidities of Korean PN patients who were treated at the Catholic Medical Center Health System dermatology outpatient clinic during the period 2007–2020. Because there is no drug approved by the US Food and Drug Administration (FDA) for PN [15], dermatologist prescriptions for PN were analyzed to identify management strategies in a real-world setting.

## 2. Materials and Methods

### 2.1. Data Source

A retrospective cross-sectional study was conducted among outpatients from the Catholic Medical Center (i.e., the largest health organization in Korea with 7 affiliated hospitals-Incheon, Yeouido, Uijeongbu, Bucheon, Eunpyeong, and Seoul St. Mary’s Hospital and St. Vincent’s Hospital). The data were extracted from the institution’s clinical data warehouse (CDW), which contains various operational data (i.e., demographics, diagnosis, prescriptions, visitation dates) of both subsidized and non-subsidized cases.

### 2.2. Ethics

The study protocol was reviewed and approved by the Catholic Medical Center Ethics Committee (XC21WIDI0011).

### 2.3. Study Population

The study population of patients that were primarily diagnosed with PN based on the *KCD* (L28.1) over a 14-year period (from 2007 to 2020) were selected from the CDW. To maximize diagnostic accuracy, subjects were limited to those diagnosed by a dermatologist at the dermatology outpatient clinic. The control population for comorbidity analyses included sex- and age (determined on 1 January 2020)-matched dermatology outpatients without PN during the same time span (1:4 matching).

### 2.4. Study Outcomes

The annual prevalence of PN (per thousand, ‰) was defined as follows: number of PN patients/number of patients who visited the dermatologic outpatient clinic at the CMC during a one-year time-period × 1000. Sample record of patients diagnosed with PN in a specific year were classified as PN patients of that year. An individual’s age was determined as that on 1 January each year, and PN patients were categorized into 10-year age groups, except for the 0–20- and 81–100-year-old groups; the two lowest and two highest age groups were merged due to the small numbers of PN samples.

The outcome of interest was concurrent systemic diseases including mental and neurological diseases (Parkinson’s disease, dementia, depression, anxiety disorder, stress disorder, schizophrenia, attention deficit hyperactivity disorder (ADHD)), vascular diseases (arterial hypertension, ischemic heart disease, cerebrovascular disease, heart failure), allergic and respiratory diseases (allergic rhinitis, atopic dermatitis, allergic conjunctivitis, asthma, chronic obstructive pulmonary disease (COPD)), autoimmune diseases (systemic lupus erythematosus (SLE), Sjogren’s syndrome, systemic sclerosis, Bechet’s disease, rheumatoid arthritis, Crohn’s disease, ulcerative colitis, ankylosing spondylitis, hyper- and hypothyroidism, autoimmune thyroiditis), cancers (Hodgkin’s lymphoma, non-Hodgkin’s lymphoma, multiple myeloma, thyroid cancer, lung cancer, gastric cancer, colorectal cancer, hepatobiliary cancer), metabolic, nutritional, and renal disease (type 2 diabetes mellitus (DM), obesity, dyslipidemia, nutritional anemia, osteoporosis, chronic kidney disease (CKD)), and infectious disorders (H. pylori infection, hepatitis B, hepatitis C, tuberculosis). Disease designations were assigned once an individual was diagnosed at least twice by a specialist during the study period (2007 to 2020). Patients were considered to have a PN prescription only when administered by a dermatologist under PN diagnosis (L28.1 as a primary diagnosis, same day).

### 2.5. Statistical Analysis

Python 3 version 3.6.7 (Python Software Foundation, DE, US) was used for basic data processing and statistical analysis. The correlation between PN prevalence (annual prevalence of each age group summed over the entire study period (2007–2020)) and age (median age of the age groups: 10, 25, 35, 45, 55, 65, 76, and 90 years) was calculated using Pearson correlation analysis. Conditional logistic regression modeling (survival package (Therneau, 2014) within the R tool (version 3.5.1)) was adopted to identify the comorbidity risk of PN and the associated subgroup analysis based on sex and age. Subgroups by sex were corrected by setting age as strata, and vice versa. Because the median and mean ages of the PN population (2007–2020) were 60 and 58.1 years, respectively, we divided our population based on 60 years of age (≥60 vs. <60 years) for subgroup analysis. Conditional logistic regression was used to analyze the prescription patterns for PN according to sex and age. *p* ≤ 0.05 was recognized as statistically significant.

## 3. Results

### 3.1. Epidemiology of PN

**Figure 1:**
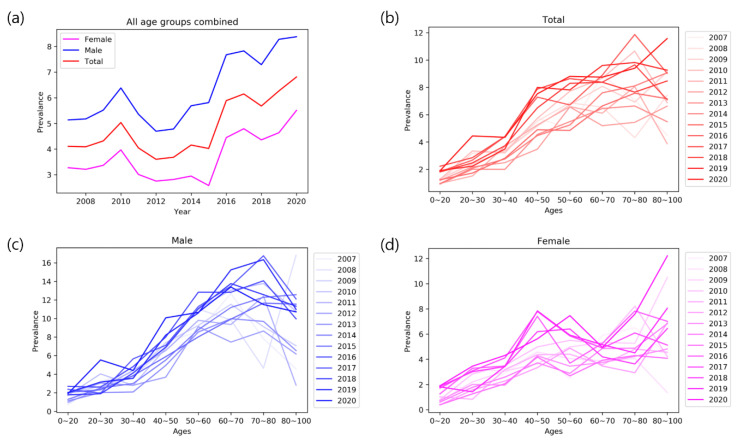
(**a**) The annual prevalence of PN per 1000 dermatology outpatient population (2007–2020) (total, male, female), (**b**) prevalence of PN (total) by age, (**c**) prevalence of PN (male) by age, and (**d**) prevalence of PN (female) by age.

The average annual prevalence of PN (per 1000 dermatology outpatient population) increased from the first half of the study period (4.11, 2007–2013) to the second (5.53, 2014–2020; *p* = 0.03) (Table 1). The annual PN prevalence was higher among males than females throughout the observation period (*p* < 0.001; Figure 1a). It also increased with age, which was a trend in both male and female populations (Figure 1b–d). A positive correlation was seen between age and prevalence of PN over 2007–2020 (R = 0.95, *p* = 0.0003), which remained significant in both sexes (females, R = 0.94, *p* = 0.0006; males, R = 0.90, *p* = 0.002).

### 3.2. Comorbidities of PN

A total of 3591 patients with PN were identified between 2007–2020. Of the 3591 PN patients, the mean age was 58.1 ± 19.4 years, and 56.8% were male (Table 2). Demographics of the control population (sex- and age-matched dermatology outpatients without PN, 1:4 matching) are shown in Table 2.

According to the conditional logistic regression analysis, individuals with PN were significantly more likely to have a number of mental and neurological diseases (dementia (adjusted odds ratio (aOR), 1.44), depression (aOR, 1.38), anxiety disorder (aOR, 1.30), stress disorder (aOR, 1.81), and schizophrenia (aOR, 2.15)), vascular diseases (arterial hypertension (aOR, 1.50), and ischemic heart disease (aOR, 1.29)), allergic and respiratory diseases (allergic rhinitis (aOR, 2.04), atopic dermatitis (aOR, 2.16), allergic conjunctivitis (aOR, 1.36), and asthma (aOR, 1.47)), autoimmune diseases (SLE (aOR, 2.02), rheumatoid arthritis (aOR, 0.66) Crohn’s disease (aOR, 2.26), hyperthyroidism (aOR, 1.60), hypothyroidism (aOR, 1.70), and autoimmune thyroiditis (aOR, 2.43)), cancer (non-Hodgkin’s lymphoma (aOR, 1.95)), metabolic, nutritional and renal diseases (type 2 DM (aOR, 1.37), dyslipidemia (aOR, 1.88), nutritional anemia (aOR, 1.38), osteoporosis (aOR, 1.67), and CKD (aOR, 1.48)), and infection (hepatitis B (aOR, 1.33)) than were controls (Table 3).

#### 3.2.1. Subgroup Analysis by Sex

Upon subgroup analysis by sex, female patients with PN were significantly more likely to experience a variety of mental and neurological diseases (dementia (aOR, 1.73), depression (aOR, 1.44), and stress disorder (aOR, 2.72)), vascular diseases (arterial hypertension (aOR, 1.33), and ischemic heart disease (aOR, 1.41)), allergic and respiratory diseases (allergic rhinitis (aOR, 2.14), atopic dermatitis (aOR, 2.18), and asthma (aOR, 1.49)), autoimmune diseases (Crohn’s disease (aOR, 3.29), and hypothyroidism (aOR, 1.91)), and metabolic, nutritional and renal disease (type 2 DM (aOR, 1.36), dyslipidemia (aOR, 1.76), osteoporosis (aOR, 1.69), and CKD (aOR, 1.65)) (Table 4).

Male subjects with PN were diagnosed frequently with a number of mental and neurological diseases (schizophrenia (aOR, 3.30)), vascular diseases (arterial hypertension (aOR, 1.64) and ischemic heart disease (aOR, 1.27)), allergic and respiratory diseases (allergic rhinitis (aOR, 1.97), atopic dermatitis (aOR, 2.15), and asthma (aOR, 1.48)), autoimmune diseases (SLE (aOR, 4.11), rheumatoid arthritis (aOR, 0.55), hyperthyroidism (aOR, 1.80), and autoimmune thyroiditis (aOR, 4.20)), cancers (non-Hodgkin’s lymphoma (aOR, 1.93) and multiple myeloma (aOR, 2.83)) and metabolic, nutritional, or renal disease (type 2 DM (aOR, 1.37), dyslipidemia (aOR, 1.98), and CKD (aOR, 1.39)) (Table 4).

#### 3.2.2. Subgroup Analysis with Age

When stratified by age, PN patients younger than 60 years experienced a number of mental and neurological diseases (anxiety disorder (aOR, 1.53), stress disorder (aOR, 1.92), and schizophrenia (aOR, 2.20)), vascular diseases (arterial hypertension (aOR, 1.33)), allergic and respiratory disease (allergic rhinitis (aOR, 2.08), atopic dermatitis (aOR, 1.89), and allergic conjunctivitis (aOR, 1.67)), and autoimmune diseases (hyperthyroidism (aOR, 2.04) and hypothyroidism (aOR, 1.97)), and metabolic, nutritional, or renal diseases (type 2 DM (aOR, 1.48), dyslipidemia (aOR, 1.79), and CKD (aOR, 1.58)) (Table 4).

PN patients 60 years of age and over were frequently diagnosed with a variety of mental and neurological diseases (dementia (aOR, 1.51), depression (aOR, 1.47)), vascular disease (arterial hypertension (aOR, 1.58) and ischemic heart disease (aOR, 1.35)), allergic and respiratory diseases (allergic rhinitis (aOR, 2.04), atopic dermatitis (aOR, 2.78), and asthma (aOR, 1.48)), autoimmune diseases (rheumatoid arthritis (aOR, 0.62), Crohn’s disease (aOR, 18.5), hypothyroidism (aOR, 1.59), and autoimmune thyroiditis (aOR, 2.66)), cancer (non-Hodgkin’s lymphoma (aOR, 2.08)), metabolic, nutritional, or renal diseases (type 2 DM (aOR, 1.35), dyslipidemia (aOR, 1.93), nutritional anemia (aOR, 1.61), osteoporosis (aOR, 1.60) and CKD (aOR, 1.44)), and infection (hepatitis B (aOR, 1.59)) (Table 4).

### 3.3. The Prescription Pattern of PN

**Figure 2:**
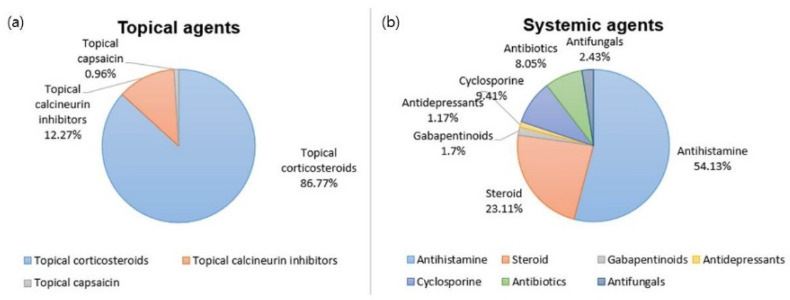
Prescription patterns for PN: (**a**) topical agents, (**b**) systemic agents.

Of the 3591 patients with PN, 2867 (79.8%) were prescribed at least one topical agent, and 3024 (84.2%) were prescribed at least one systemic agent to control PN. Among the topical agents, steroids (86.8%) were prescribed most frequently, followed by calcineurin inhibitors (12.3%) and capsaicin (0.96%) (Figure 2a). With regard to systemic agents, antihistamines (54.1%) were used most frequently, followed by oral steroids (23.1%), cyclosporin (9.41%), antibiotics (8.05%), antifungals (2.43%), gabapentinoids (1.70%), and antidepressants (1.17%) (Figure 2b). Other than topical and oral agents, 21.7% of the PN population received intralesional steroid injections, and 6.20% underwent phototherapy for disease management.

According to subgroup analysis (age, sex), PN patients 60 years of age and over were more likely to receive topical steroids (aOR, 1.17; 95% CI, 1.04–1.31) and less likely to be prescribed oral steroids (OR, 0.78, 95% CI, 0.70–0.87) and antibiotics (OR, 0.78; 95% CI, 0.66–0.92) than were those younger than 60 years (Table 5).

## 4. Discussion

Based on the current data, the estimated annual prevalence of PN in Korea is 4.82 cases per 1000 dermatology outpatients. Considering that our population is limited to dermatology outpatients, it is understandable that the number is higher than the nationwide annual prevalence of PN reported from Poland (6.52 cases per 100,000 population) [8], Germany (40 cases per 100,000 population) [9], and the US (72 cases per 100,000 population) [10].

Our long-term, real-world dataset suggests an increasing trend in the annual prevalence of PN (4.11, 2007–2013 vs. 5.53, 2014–2020), which is likely due to the longer lifespan (supported by the positive correlation shown between age and prevalence of PN) and increased awareness of the disease.

The mean age of our PN subjects was 58.1 years, which is similar to the findings from previous studies (61.5 years; Poland [8], 50.9 years; US [10]). A predominance in females was noted in the Caucasian and African American PN population in the US [11]; however, the sex ratio of our PN patients was male dominant (56.8%), which matches the findings from Boozalis et al. [11] in Asians (58.1% male, 41.9% female).

Our patient population had increased likelihood of carrying mental and neurologic, vascular, allergic and respiratory, autoimmune, cancer, metabolic, nutritional, and renal disease diagnoses, which emphasizes the increased disease burden associated with PN relative to other skin conditions.

Psychiatric health conditions have been linked not only with PN [10,11,16,17] but also with skin diseases such as atopic dermatitis and psoriasis [18,19]. The increased presence of schizophrenia, anxiety, depression, and other conditions in our study patients compared to dermatologic outpatients stresses a psychological component to PN as well as the strong impact of PN on mental wellbeing [20]. Our PN population also had a higher prevalence of systemic illnesses and non-Hodgkin’s lymphoma. The associations between PN and CKD, arterial hypertension, and ischemic heart disease in excess to the risk in other skin conditions supports the systemic nature of PN and is in line with findings from previous studies [10,11]. Our data also support prior reports that indicated an association of PN with hepatitis B infection and non-Hodgkin’s lymphoma, as well as with type 2 DM, thyroid disease, dyslipidemia, anemia, and osteoporosis [10,11,12,21,22]. Interestingly, the patients with PN in our study were less likely to have rheumatoid arthritis, which was observed in African Americans with PN compared to those with psoriasis (OR, 0.3; 95% CI, 0.1–0.8) [11]. Finally, the heightened likelihood of allergic conditions in our PN population can be accredited to the patient subset who were atopic. Allergic susceptibility is recognized in PN, particularly in early-onset PN [5,23].

In addition to confirming previous links between PN and comorbidities, our subgroup analyses showed that female patients and individuals 60 years of age and over experienced dementia and depression, while only males and patients 60 and over had heightened risk of non-Hodgkin’s lymphoma. Accordingly, there is need for a heightened index for clinicians to suspect and diagnose patients and for appropriate screening for PN patients to account for a patient’s race, age, and sex.

Analysis of prescription patterns for PN in Korea indicated that steroids and calcineurin inhibitors were the two most frequent topical treatments, both of which address the immunologic component of PN [6]. These were followed by topical capsaicin, which modulates the neural component of PN [6]. It is likely that topical capsaicin is less preferred due to reported intense burning and irritation associated with its use.

Korean dermatologists most frequently prescribed oral antihistamines for PN, followed by oral steroids. This differs drastically from the treatment pattern in the US [24], where oral antihistamines are prescribed rarely. The discrepancy likely is related to the medical insurance system in Korea, which is controlled strictly by the Korean government. The use of oral medications other than antihistamines and steroids (oral cyclosporin, gabapentinoids, antidepressants, methotrexate) for PN are restricted, leaving limited options. Oral antihistamines were recommended as a first-line treatment in a review paper on PN (evidence level IV) [25], but there is insufficient evidence for its use in PN [26].

International experts do not recommend systemic steroids for PN (evidence level VI) [25], but it is frequently prescribed both in Korea and the US [24] in real-world settings. The analysis from this study indicated that dermatologists in Korea are more cautious with prescribing systemic steroids in the elderly population (60 years and over) who are more prone to developing complications.

Recently, a number of newer drugs have been introduced and are under clinical trials. The IFSI (International Forum for the Study of Itch) guideline in 2020 has recommended opioid modulators, biologics, and small molecules as promising agents for the treatment of PN [27]. Although we have limited experience with these agents in Korean PN, a bright future lies ahead.

## 5. Strengths and Limitations

This study analyzed real-world Korean PN data utilizing a large cohort of dermatology outpatients in Korea and is the first and only study to describe the epidemiology, comorbidities, and prescription patterns of PN in Korea. Study limitations include the retrospective analysis of registry data, where information on the severity of PN and lifestyle risk factors is not available. Our data also showed that PN was associated with conditions and comorbidities, but we were unable to establish causality. Furthermore, due to the rarity of HIV, we were not able to check the association between HIV and incidence of PN in Korean patients.

## 6. Conclusions

Overall, our data show that PN is an important disease among Korean dermatology outpatients with a high comorbidity burden compared to dermatology outpatients without PN. Korean patients with PN suffer not only due to the debilitating nature of the disease, but also due to the lack of effective treatment which may be resolved with use of the promising new drugs (i.e., opioid modulators, biologics, and small molecules). Further research is warranted to better understand the pathogenesis, identify risk factors associated with PN, and employ optimal care.

## Figures and Tables

**Table 1:** Prevalence of PN per 1000 dermatology outpatient population.

**Year/Age**	**2007**	**2008**	**2009**	**2010**	**2011**	**2012**	**2013**	**2007–2013**
81–100	4.47	7.37	9.05	6.83	3.86	6.58	5.46	6.23
71–80	7.02	4.30	6.87	10.54	7.99	5.41	6.59	6.96
61–70	8.82	6.51	8.01	8.60	6.07	5.17	6.41	7.08
51–60	4.98	6.83	6.68	7.66	6.54	6.53	5.46	6.38
40–50	5.71	5.50	5.15	5.70	5.22	3.47	4.53	5.04
31–40	3.39	3.34	3.34	3.20	3.68	2.49	2.00	3.06
21–30	2.33	3.17	2.41	3.34	1.51	2.18	2.01	2.42
0–20	1.29	1.17	1.33	1.27	0.97	1.19	0.92	1.16
Total	4.09	4.07	4.30	5.01	4.03	3.59	3.67	4.11
**Year/Age**	**2014**	**2015**	**2016**	**2017**	**2018**	**2019**	**2020**	**2014–2020**
81–100	8.99	7.12	8.95	7.04	8.40	9.17	11.44	8.73
71–80	8.04	7.51	11.73	9.56	7.59	9.72	9.31	9.07
61–70	7.54	6.59	8.81	8.31	8.30	9.51	8.67	8.25
51–60	5.22	4.83	6.67	8.56	8.22	7.75	8.73	7.14
40–50	4.45	4.88	7.23	7.81	6.46	7.94	7.47	6.60
31–40	2.75	2.81	4.38	4.39	3.71	3.48	4.33	3.69
21–30	2.08	1.73	2.67	2.85	2.47	2.23	4.43	2.64
0–20	0.96	1.24	1.83	2.22	1.81	1.91	1.89	1.69
Total	4.14	4.01	5.85	6.11	5.65	6.23	6.76	5.53

Data are presented in ‰.

**Table 2:** Demographic characteristics of the PN and control populations.

Variables	PN (*n* = 3591)	Control (*n* = 14,364)
Sex		
Female, *n* (%)	1551 (43.2)	6204 (43.2)
Male, *n* (%)	2040 (56.8)	8160 (56.8)
Age (years)	58.1 ± 19.4	56.8 ± 19.4
0–20, *n* (%)	159 (4.43)	636 (4.43)
21–30, *n* (%)	198 (5.51)	792 (5.51)
31–40, *n* (%)	327 (9.11)	1308 (9.91)
41–50, *n* (%)	451 (12.6)	1804 (12.6)
51–60, *n* (%)	672 (18.7)	2688 (18.7)
61–70, *n* (%)	780 (21.7)	3120 (21.7)
71–80, *n* (%)	578 (16.1)	2312 (16.9)
81–100, *n* (%)	426 (11.9)	1704 (11.9)

**Table 3:** Association between PN and various systemic diseases.

Variables	Number of Cases		OR	95% CI	*p*-Value	aOR *	95% CI	*p*-Value
	PN	Control						
***Mental and neurologic diseases***								
Parkinson’s disease	18	80	0.89	0.53~1.50	0.68	0.87	0.54~1.41	0.59
Dementia	47	68	2.78	1.91~4.05	<0.0001	1.44	1.05~1.96	0.02
Depression	126	214	2.40	1.92~3.01	<0.0001	1.38	1.14~1.67	0.0009
Anxiety disorder	70	98	2.89	2.12~3.94	<0.0001	1.30	1.02~1.67	0.03
Stress disorder	28	33	3.41	2.05~5.65	<0.0001	1.81	1.24~2.65	0.002
Schizophrenia	16	14	4.58	2.23~9.40	<0.0001	2.15	1.30~3.55	0.002
ADHD	2	3	2.66	0.44~15.97	0.28	1.38	0.33~5.65	0.65
***Vascular diseases***								
Hypertension (arterial)	225	409	2.28	1.92~2.69	<0.0001	1.50	1.30~1.73	<0.0001
Ischemic heart disease	270	547	2.05	1.76~2.38	<0.0001	1.29	1.13~1.48	0.0001
Cerebrovascular disease	207	650	1.29	1.09~1.51	0.001	0.92	0.80~1.07	0.33
Heart failure	70	122	2.32	1.72~3.12	<0.0001	1.26	0.98~1.62	0.06
***Allergic and respiratory diseases***								
Allergic rhinitis	134	133	4.14	3.25~5.28	<0.0001	2.04	1.70~2.45	<0.0001
Atopic dermatitis	311	399	3.31	2.84~3.86	<0.0001	2.16	1.91~2.45	<0.0001
Allergic conjunctivitis	63	104	2.44	1.78~3.35	<0.0001	1.36	1.04~1.76	0.02
Asthma	86	105	3.33	2.49~4.44	<0.0001	1.47	1.18~1.85	0.006
COPD	78	183	1.72	1.31~2.24	0.0001	1.15	0.91~1.46	0.22
***Autoimmune diseases***								
SLE	10	10	4.00	1.66~9.63	0.001	2.02	1.07~3.81	0.02
Sjogren’s syndrome	8	33	0.96	0.44~2.10	0.93	0.83	0.41~1.68	0.6
Systemic sclerosis	2	10	0.79	0.17~3.65	0.77	0.76	0.18~3.05	0.69
Behcet’s disease	5	21	0.95	0.35~2.52	0.92	0.89	0.36~2.14	0.79
Rheumatoid arthritis	42	240	0.69	0.50~0.96	0.03	0.66	0.48~0.89	0.008
Crohn’s disease	8	11	2.91	1.17~7.24	0.02	2.26	1.11~4.61	0.02
Ulcerative colitis	4	34	0.47	0.16~1.32	0.15	0.43	0.16~1.18	0.10
Ankylosing spondylitis	6	22	1.09	0.44~2.69	0.85	1.12	0.50~2.50	0.77
Hyperthyroidism	31	66	1.88	1.22~2.89	0.003	1.60	1.12~2.28	0.009
Hypothyroidism	43	64	2.70	1.83~3.99	<0.0001	1.70	1.25~2.31	0.0007
Autoimmune thyroiditis	14	9	6.24	2.69~14.43	<0.0001	2.43	1.42~4.16	0.001
***Cancers***								
Hodgkin’s lymphoma	1	2	2.00	0.18~22.06	0.57	1.01	0.13~7.72	0.98
Non-Hodgkin’s lymphoma	20	31	2.58	1.47~4.54	0.0009	1.95	1.23~3.07	0.004
Multiple myeloma	4	13	1.23	0.40~3.77	0.71	1.31	0.48~3.51	0.59
Thyroid cancer	30	97	1.23	0.82~1.86	0.3	1.04	0.72~1.50	0.80
Lung cancer	34	163	0.83	0.57~1.20	0.33	0.89	0.63~1.25	0.51
Gastric cancer	26	183	0.56	0.37~0.85	0.006	0.68	0.46~1.01	0.06
Colorectal cancer	41	145	1.13	0.79~1.60	0.48	1.05	0.77~1.44	0.72
Hepatobiliary cancer	43	134	1.28	0.91~1.81	0.152	1.06	0.78~1.44	0.69
***Metabolic, nutritional, and renal diseases***								
Type 2 DM	389	817	2.01	1.77~2.28	<0.0001	1.37	1.22~1.54	<0.0001
Obesity	10	53	0.75	0.38~1.48	0.41	0.66	0.36~1.24	0.2
Dyslipidemia	115	158	2.97	2.33~3.79	<0.0001	1.88	1.56~2.27	<0.0001
Nutritional anemia	41	71	2.32	1.58~3.42	<0.0001	1.38	1.01~1.90	0.04
Osteoporosis	62	85	2.95	2.12~4.10	<0.0001	1.67	1.29~2.18	0.0001
CKD	258	427	2.52	2.15~2.96	<0.0001	1.48	1.29~1.70	<0.0001
***Infections***								
*H.pylori* infection	11	29	1.51	0.75~3.04	0.23	1.28	0.70~2.34	0.40
Hepatitis B	55	146	1.51	1.10~2.06	0.009	1.33	1.01~1.74	0.03
Hepatitis C	17	61	1.11	0.65~1.91	0.69	0.83	0.51~1.35	0.46
Tuberculosis	37	83	1.79	1.21~2.64	0.003	1.32	0.95~1.84	0.09

Abbreviations: ADHD, attention deficit hyperactivity disorder; CI, confidence interval; COPD, chronic obstructive pulmonary disease; CKD, chronic kidney disease; DM, diabetes mellitus; H.pylori, Helicobacter pylori; NA, not available; OR, odds ratio; SLE, systemic lupus erythematosus. * Adjusted by age and sex.

**Table 4:** Association between PN and various systemic diseases.

Variables	Sex						Age					
	Male			Female			<60			≥60		
	aOR *	95% CI	*p*-Value	aOR *	95% CI	*p*-Value	aOR *	95% CI	*p*-Value	aOR *	95% CI	*p*-Value
***Mental and neurologic diseases***												
Parkinson’s disease	1.06	0.58~1.95	0.83	0.76	0.36~1.64	0.49	N/A			0.89	0.56~1.44	0.65
Dementia	1.24	0.80~1.95	0.33	1.73	1.11~2.72	0.01	N/A			1.51	1.10~2.08	0.01
Depression	1.32	0.98~1.79	0.06	1.44	1.13~1.86	0.003	1.30	0.93~1.82		1.47	1.16~1.87	0.00
Anxiety disorder	1.24	0.86~1.80	0.24	1.33	0.94~1.89	0.10	1.53	1.04~2.25		1.14	0.82~1.60	0.43
Stress disorder	1.36	0.80~2.33	0.26	2.72	1.60~4.65	0.0002	1.92	1.18-3.13	0.008	1.66	0.88~3.15	0.11
Schizophrenia	3.30	1.64~6.65	0.0008	1.60	0.78~3.28	0.19	2.20	1.28~3.79	0.004	1.64	0.40~6.76	0.48
ADHD	1.53	0.36~6.51	0.55	N/A			1.08	0.25~4.57	0.91	N/A		
***Cardiovascular diseases***												
Hypertension (arterial)	1.64	1.36~1.98	<0.0001	1.33	1.07~1.68	0.01	1.33	1.02~1.74	0.03	1.58	1.34~1.88	<0.0001
Ischemic heart disease	1.27	1.08~1.51	0.003	1.41	1.11~1.80	0.005	1.01	0.73~1.38	0.94	1.35	1.17~1.59	0.0001
Cerebrovascular disease	0.85	0.71~1.03	0.09	1.12	0.87~1.46	0.35	0.96	0.65~1.43	0.87	0.92	0.79~1.09	0.35
Heart failure	1.20	0.85~1.71	0.3	1.25	0.87~1.80	0.22	1.02	0.51~2.03	0.94	1.30	1.00~1.71	0.05
***Allergic and respiratory diseases***												
Allergic rhinitis	1.97	1.52~2.58	<0.0001	2.14	1.66~2.77	<0.0001	2.08	1.63~2.66	<0.0001	2.04	1.55~2.70	<0.0001
Atopic dermatitis	2.15	1.84~2.51	<0.0001	2.18	1.79~2.66	<0.0001	1.89	1.62~2.20	<0.0001	2.78	2.27~3.42	<0.0001
Allergic conjunctivitis	1.46	0.99~2.17	0.05	1.27	0.89~1.83	0.17	1.67	1.19~2.35	0.002	0.98	0.65~1.50	0.95
Asthma	1.48	1.07~2.06	0.01	1.49	1.09~2.04	0.01	1.52	1.10~2.11	0.01	1.48	1.08~2.05	0.01
COPD	1.13	0.87~1.47	0.34	1.53	0.83~2.86	0.17	1.77	0.92~3.39	0.08	1.10	0.85~1.42	0.45
***Autoimmune diseases***												
SLE	4.11	0.99~16.92	0.04	1.59	0.77~3.30	0.21	2.01	0.88~4.57	0.09	2.28	0.82~6.42	0.11
Sjogren’s syndrome	0.95	0.13~6.84	0.96	0.83	0.39~1.78	0.63	0.97	0.39~2.39	0.94	0.62	0.20~1.97	0.41
Systemic sclerosis	0.99	0.14~7.06	0.99	0.59	0.08~4.28	0.6	0.87	0.12~6.24	0.89	0.71	0.10~5.16	0.73
Behcet’s disease	0.90	0.23~3.63	0.88	0.88	0.29~2.78	0.84	0.61	0.15~2.48	0.49	1.31	0.42~4.08	0.63
Rheumatoid arthritis	0.55	0.32~0.94	0.02	0.70	0.48~1.04	0.07	0.71	0.43~1.16	0.17	0.62	0.42~0.94	0.02
Crohn’s disease	1.62	0.58~4.53	0.35	3.29	1.22~8.88	0.01	2.05	0.96~4.38	0.06	18.5	1.09~314.6	0.04
Ulcerative colitis	0.37	0.12~1.19	0.09	1.39	0.19~10.05	0.73	0.29	0.07~1.21	0.09	0.50	0.07~3.63	0.50
Ankylosing spondylitis	0.85	0.32~2.29	0.75	3.12	0.77~12.65	0.11	1.10	0.45~2.68	0.81	1.33	0.19~9.49	0.77
Hyperthyroidism	1.80	1.04~3.14	0.03	1.55	0.97~2.49	0.06	2.04	1.23~3.38	0.005	1.32	0.80~2.22	0.27
Hypothyroidism	1.21	0.64~2.32	0.55	1.91	1.34~2.72	0.0003	1.97	1.19~3.24	0.007	1.59	1.08~2.38	0.02
Autoimmune thyroiditis	4.20	1.85~9.55	0.0006	1.81	0.89~3.73	0.1	1.59	0.50~4.97	0.42	2.66	1.42~4.99	0.002
***Cancers***												
Hodgkin’s lymphoma	0.89	0.11~7.19	0.91	N/A			N/A			0.89	0.12~6.90	0.91
Non-Hodgkin’s lymphoma	1.93	1.13~3.31	0.01	2.03	0.84~4.94	0.11	1.46	0.46~4.59	0.51	2.08	1.27~3.44	0.003
Multiple myeloma	2.83	1.06~7.62	0.03	N/A			3.32	0.43~25.24	0.24	1.09	0.35~3.42	0.87
Thyroid cancer	1.26	0.67~2.37	0.47	0.94	0.61~1.48	0.81	1.11	0.68~1.81	0.65	0.96	0.56~1.69	0.913
Lung cancer	1.04	0.72~1.53	0.8	0.54	0.24~1.22	0.13	0.21	0.03~1.53	0.12	1.00	0.71~1.43	0.96
Gastric cancer	0.73	0.48~1.12	0.15	0.54	0.20~1.46	0.22	0.39	0.09~1.59	0.19	0.72	0.49~1.09	0.12
Colorectal cancer	1.02	0.71~1.48	0.89	1.09	0.60~1.99	0.77	0.81	0.33~1.95	0.64	1.09	0.79~1.54	0.57
Hepatobiliary cancer	1.04	0.73~1.51	0.79	1.27	0.72~2.29	0.4	1.05	0.50~2.21	0.88	1.05	0.75~1.49	0.75
***Metabolic, nutritional and renal disease***												
Type 2 DM	1.37	1.19~1.59	<0.0001	1.36	1.13~1.66	0.001	1.48	1.18~1.85	0.0005	1.35	1.18~1.55	<0.0001
Obesity	1.19	0.53~2.68	0.66	0.40	0.15~1.07	0.06	0.76	0.38~1.54	0.46	0.48	0.12~2.01	0.32
Dyslipidemia	1.98	1.53~2.57	<0.0001	1.76	1.33~2.34	0.0001	1.79	1.30~2.45	0.0003	1.93	1.52~2.46	<0.0001
Nutritional anemia	1.68	0.96~2.95	0.06	1.29	0.87~1.91	0.19	1.12	0.69~1.84	0.62	1.61	1.06~2.45	0.02
Osteoporosis	1.27	0.59~2.74	0.53	1.69	1.27~2.25	0.0003	1.07	0.39~2.91	0.88	1.6	1.22~2.12	0.0009
CKD	1.39	1.17~1.66	0.0001	1.65	1.30~2.11	<0.0001	1.58	1.20~2.10	0.001	1.44	1.23~1.70	<0.0001
***Infection***												
H. pylori infection	1.81	0.84~3.90	0.12	0.85	0.32~2.31	0.76	1.19	0.48~2.93	0.69	1.33	0.59~2.99	0.47
Hepatitis B	1.34	0.95~1.89	0.09	1.26	0.81~1.99	0.30	0.96	0.59~1.54	0.87	1.59	1.14~2.24	0.006
Hepatitis C	0.93	0.53~1.67	0.82	0.68	0.28~1.65	0.39	0.74	0.30~1.82	0.52	0.83	0.46~1.52	0.56
Tuberculosis	1.28	0.85~1.96	0.23	1.42	0.83~2.47	0.20	1.63	0.86~3.10	0.12	1.27	0.87~1.88	0.21

* Adjusted by age and sex. Abbreviations: ADHD, attention deficit hyperactivity disorder; CI, confidence interval; COPD, chronic obstructive pulmonary disease; CKD, chronic kidney disease; DM, diabetes mellitus; H. pylori, Helicobacter pylori; NA, not available; OR, odds ratio; SLE, systemic lupus erythematosus.

**Table 5:** Prescription patterns for PN.

Variables	Sex (Case/Reference: Male/Female)			Age (Years) (Case/Reference: ≥60/<60 Years)		
	aOR *	95% CI	*p*-Value	aOR *	95% CI	*p*-Value
***Topical agents***						
Corticosteroid	1.05	0.94~1.17	0.32	1.17	1.04~1.31	0.008
CNI	0.92	0.79~1.07	0.30	0.98	0.83~1.16	0.87
Capsaicin	0.82	0.47~1.42	0.47	1.50	0.97~2.30	0.06
***Systemic agents***						
Antihistamines	0.99	0.87~1.12	0.93	1.10	0.96~1.26	0.13
Oral steroid	0.95	0.86~1.05	0.36	0.78	0.70~0.87	<0.0001
Gabapentin	0.96	0.72~1.27	0.78	1.30	0.99~1.71	0.05
Antidepressants	0.83	0.58~1.19	0.32	1.21	0.87~1.69	0.24
Cyclosporine	1.08	0.94~1.23	0.23	0.88	0.76~1.03	0.11
Antibiotics	1.07	0.93~1.23	0.29	0.78	0.66~0.92	0.003
Antifungals	1.23	0.99~1.54	0.05	1.15	0.89~1.47	0.26
***Other Therapies***						
Phototherapy	0.98	0.82~1.18	0.89	1.13	0.93~1.36	0.19
Intralesional injection with corticosteroids	1.05	0.94~1.16	0.35	1.02	0.91~1.15	0.62

* Adjusted by age and sex. Abbreviations: CI, confidence interval; CNI, calcineurin inhibitor; OR, odds ratio.

## Data Availability

Data available on request due to restrictions. The data presented in this study are available on request from the corresponding author.
